# Signal generation in dynamic interferometric displacement detection

**DOI:** 10.3762/bjnano.15.87

**Published:** 2024-08-20

**Authors:** Knarik Khachatryan, Simon Anter, Michael Reichling, Alexander von Schmidsfeld

**Affiliations:** 1 Institut für Physik, Universität Osnabrück, Barbarastr. 7, 49076 Osnabrück, Germany

**Keywords:** amplitude calibration, displacement detection, force microscopy, interferometer signal, NC-AFM

## Abstract

Laser interferometry is a well-established and widely used technique for precise displacement measurements. In a non-contact atomic force microscope (NC-AFM), it facilitates the force measurement by recording the periodic displacement of an oscillating microcantilever. To understand signal generation in a NC-AFM-based Michelson-type interferometer, we evaluate the non-linear response of the interferometer to the harmonic displacement of the cantilever in the time domain. As the interferometer signal is limited in amplitude because of the spatial periodicity of the interferometer light field, an increasing cantilever oscillation amplitude creates an output signal with an increasingly complex temporal structure. By the fit of a model to the measured time-domain signal, all parameters governing the interferometric displacement signal can precisely be determined. It is demonstrated, that such an analysis specifically allows for the calibration of the cantilever oscillation amplitude with 2% accuracy.

## Introduction

Optical interferometry is a reliable technique utilizing light waves to measure distance and displacement with high precision [1–2]. With the light wavelength as the length standard, a highly stable interferometer can detect displacements with an accuracy far beyond nanometer resolution [3], where the final physical limit is set by the photon emission statistics of the light source [4]. In non-contact atomic force microscopy (NC-AFM), interferometry is used to measure the periodic displacement of a (quasi) harmonically oscillating microcantilever, acting as one mirror of the interferometer, while the second mirror is the even surface of an optical fiber delivering the light to the microcantilever [5–10].

**Figure 1:**
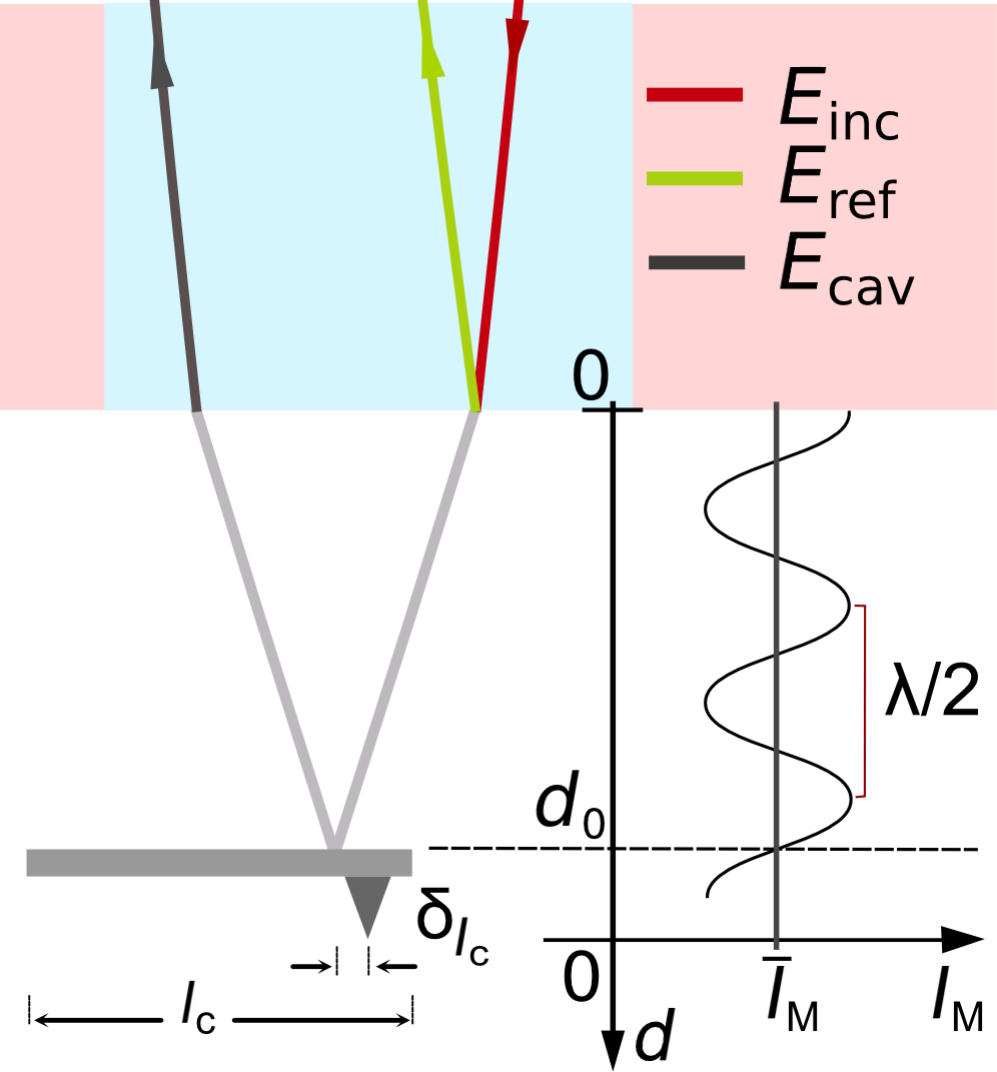
Michelson-type interferometer formed by an optical fiber end and a microcantilever. Graph and physical quantities are explained in the text.

As illustrated in Figure 1, interference occurs in the optical fiber between the light beams reflected from the fiber end (reference beam) and the cantilever (cavity beam), creating a standing wave pattern in the fiber with a spatial periodicity given by the light wavelength λ and a phase ϕ determined by the distance *d* between the fiber end and the cantilever. Any variation in *d* results in a variation of the intensity *I*_M_ recorded by a detector placed at a fixed distance to the fiber end [11]. In our setup, there is a strong imbalance of reflectivity coefficients between fiber (*r*_f_) and cantilever (*r*_c_), yielding an interferometer signal with a large average 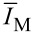 and a small intensity variation upon a change in *d*.

As light exits the fiber with a certain divergence, and the fiber core has a small diameter (4 μm), there is a finite number of multiple reflections between the cantilever and fiber. At large distance *d*, this number is small, and the setup basically acts as a Michelson interferometer. Experiments reported here are performed with the dielectric/vacuum interface of the bare fiber end acting as the first mirror and a metal-coated silicon cantilever as the second mirror. We keep the fiber–cantilever distance *d* always large enough to work in the Michelson regime characterized by a low Fabry–Pérot enhancement factor [12].

To obtain a model description of the interference light intensity at the detector, we virtually place the detector inside the fiber at its end and consider the electric field of the incident light beam *E*_inc_ at this position, the electric field of the reference light beam *E*_ref_ = *r*_f_*E*_inc_, and the electric field reflected from the cantilever and entering the fiber 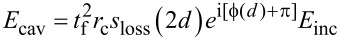. As interference occurs in the fiber, the relevant transmissivity is 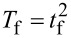, and it depends on the polarisation for (quasi) normal incidence. The a priori unknown function *s*_loss_(2*d*) describes the loss of light in the gap between the fiber end and the cantilever due to beam divergence. The spatial variation of the electric field strength due to interference is governed by the path difference 2*d* determining the phase of the interference electric fields 
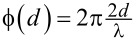
.

Linear superposition of reference and cavity beams yields, as the intensity measured at the detector position,


[1]

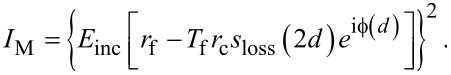



By introducing the incoming light intensity 
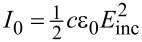
, where *c* is the speed of light in vacuum and ε_0_ is the vacuum permittivity, and the reflectivities *R*_f_ = (*r*_f_)^2^, *R*_c_ = (*r*_c_)^2^ and cavity loss *S*_loss_(2*d*) = (*s*_loss_(2*d*))^2^, Equation 1 is transformed into


[2] $$\begin{array}{c} I_{\text{M}}\left(d\right)=I_{0}[R_{\text{f}}+{\left(1-R_{\text{f}}\right)}^{2}R_{\text{c}}S_{\text{loss}}\left(2d\right)\\ -2\sqrt{R_{\text{f}}R_{\text{c}}}\left(1-R_{\text{f}}\right)\cdot \sqrt{S_{\text{loss}}\left(2d\right)}\cos\left(2\pi \frac{2d}{\lambda }\right)], \end{array}$$

where the transmissivity *T*_f_ is substituted by 1 − *R*_f_, representing the law of energy conservation. A sketch of the intensity measured at the detector of the Michelson-type interferometer *I*_M_ as a function of *d* is shown in the right part of Figure 1, where the distance dependence *S*_loss_(2*d*) has been neglected. The interference pattern has a periodicity of λ/2, while the curve crosses the mean value of intensity 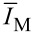 every *n*λ/4, where *n* is a positive integer. Usually, the interferometer is adjusted to positions *d*_0_ = *m*λ/8, where *m* is an odd integer representing inflection points of the interference curve, where the slope of *I*_M_(*d*) is a maximum. Such an adjustment facilitates a most sensitive displacement detection. Note, that it is not possible to adjust the interferometer to *d*_0_ with a small number *m* because of limitations in positioning the fiber end face parallel to the cantilever surface.

Upon excitation, the freely oscillating cantilever exhibits a harmonic displacement *q*(*t*) as a function of time. If a tip–surface force *F*_ts_ is present, this will introduce a slight anharmonicity, and there will be a static displacement *q*_s_ [13]. Within the harmonic approximation, which is well justified for small tip–surface forces, the cantilever displacement is [13]:


[3] $$q\left(t\right)=q_{\text{s}}+A\cdot \sin\left(2\pi f_{\text{exc}}t\right),$$

where *A* is the cantilever oscillation amplitude and *f*_exc_ is the excitation frequency kept at the resonance frequency of the cantilever for frequency-modulation NC-AFM. Further taking into account that the interferometer may be misaligned by the amount *d*_err_, we find for the time-dependent fiber-cantilever distance:


[4] $$d\left(t\right)=d_{0}+d_{\text{err}}-q\left(t\right)=d_{0}+d_{\text{err}}-q_{\text{s}}-A\cdot \sin\left(2\pi f_{\text{exc}}t\right).$$

Combining Equation 2 and Equation 4 yields the time dependence of the light intensity at the detector. As the detector measures the total incident light power, we introduce the circular illuminated effective area of the detector 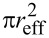. The factor *f*_loss_ takes all optical losses into account occurring in the fiber delivering the light to the cantilever and to the detector. The time domain signal of the interferometer is then given as


[5]

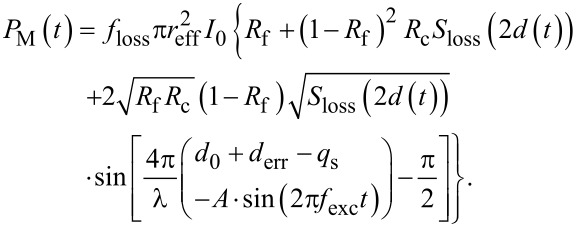



Analyzing the result, we find that the characteristics of the oscillatory part of *P*_M_ is determined by the ratio between the cantilever oscillation amplitude *A* and the wavelength λ. For *A* ≪ λ/8, the detector signal oscillates quasi-sinusoidal with the fundamental frequency *f*_exc_; for *A* ≈ λ/8, the signal is a strongly distorted sine and when increasing the amplitude further, the signal is more and more dominated by higher-frequency oscillations. Exemplary waveforms are shown schematically below in Figure 4.

## Results and Discussion

**Figure 2:**
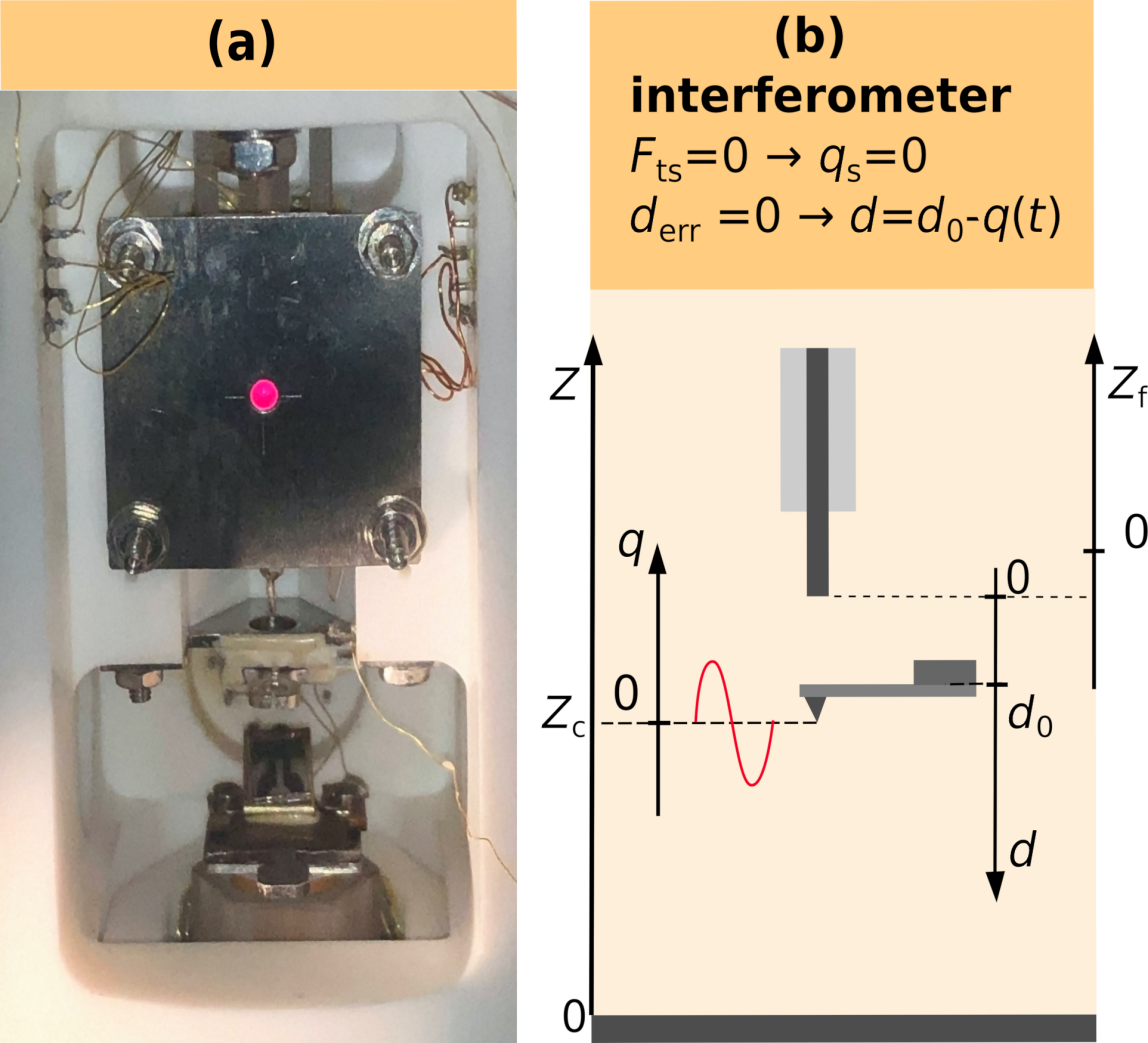
(a) Photo of the AFM scanhead showing the fiber and fiber coarse approach assembly (top), the removable cantilever holder (middle) and the sample plate with a mirror inserted for inspection purposes (bottom). (b) Coordinates for fiber movement *z*_f_, fiber cantilever distance *d* and cantilever displacement *q* in relation to the tip–sample coordinate *z* [18]. The cantilever is shown in its relaxed position where the static displacement *q*_s_ = 0 and *A* sin(2π*f*_exc_*t*) = 0. Note, that the origin of the *d*-axis is fixed at the fiber end. The interferometer yields maximum sensitivity for *d* = *d*_0_ as explained in the text.

The interferometer used for our experiments is part of a custom-built NC-AFM, operated under ultrahigh-vacuum (UHV) conditions [14]. The cantilever is a highly reflective (*R*_c_ = 0.81) aluminum-coated silicon microcantilever (type PPP-NCLR, NanoWorld AG, Neuchâtel, Switzerland) having dimensions of (225 ± 10) μm × (38 ± 8) μm × (7 ± 1) μm according to the specification of the manufacture. Using our standard procedure [15], we determined the eigenfrequency as *f*_0_ = 169.67622 kHz and a quality factor of *Q* = 9000. After transfer of the cantilever, which is glued to a cantilever holder, the cantilever is mechanically firmly attached to the AFM scan head, while the optical fiber and the sample are approached to the cantilever and the tip by piezoelectric motors for coarse motion [16] and tube piezos [17] for fine positioning in all directions. The scanhead with cantilever, sample support, and the respective motion elements are shown in Figure 2a. The fine adjustment of *d* is accomplished by the fiber tube piezo, which is in its relaxed position at *z*_f_ = 0, according to the coordinate system given in Figure 2b. Note, that the tube piezo allows for an adjustment of *d* with high accuracy; however, the absolute distance between the fiber end and the cantilever can practically neither be set nor measured. The interferometer is adjusted to a fairly large value *d*_0_ to assure operation in the Michelson mode resulting in a detector signal *I*_M_ that is much smaller than what could be obtained by working in the Fabry–Pérot mode [12].

A temperature- and intensity-stabilized laser diode light source (type 48TA-1-42037, Schäfter + Kirchhoff GmbH, Hamburg, Germany) operating at a vacuum wavelength of λ = 796.42 nm delivers the light to the cantilever via a single-mode optical fiber (type Hi780, Corning Inc., New York, USA) with a core having a refractive index of *n*_f_ = 1.45 and 4 μm diameter. Before entering the UHV system, the light passes a 3 dB beam splitter, where it is divided into two beams with almost identical power. The first part is directed to a power meter for control purposes, while the second part is guided to the interferometer in the UHV [11]. The fiber end in the interferometer is carefully cleaved to achieve high optical quality for the dielectric/vacuum interface having a reflectivity of *R*_f_ = 0.04. The fourth end of the 3 dB coupler is connected to the detector, which is a photoreceiver (model HBPR-200M-30K-SI-FC, FEMTO Messtechnik, Berlin, Germany) converting the incoming light power into a voltage signal. The photoreceiver allows for high-sensitivity low-noise measurements of DC and AC signals with a bandwidth of 200 MHz.

The interferometer is precisely aligned via a tube piezo controlled by the R9 control system (RHK Technology Inc., Troy, MI, USA). Cantilever excitation with a sine wave voltage with a well-defined amplitude *V*_exc_ and overall experiment control is accomplished by a HF2LI (Zurich Instruments, Zürich, Switzerland). Experiments are performed with the freely oscillating cantilever. Therefore, the cantilever excitation frequency *f*_exc_ is set to the eigenfrequency of the cantilever, which is determined by taking a resonance curve before each experiment. By temperature stabilisation of the laboratory and the scan head, care is taken to avoid any thermal drift of the cantilever eigenfrequency that might compromise measurements. A model MDO3000 oscilloscope (Tektronix Inc., Beaverton, OR, USA) is used to record the AC output signal of the photoreceiver, *V*_sig_, which is a voltage between 0 and 10 m*V*_pp_ with a typical noise level of less than 150 μ*V*_RMS_. Time traces with a length of 4 μs at a sampling rate of 250 MS/s are taken and quantized with a resolution of 10 bits. Each experiment comprises a set of 20 to 30 measurements with the excitation voltage amplitude *V*_exc_ ramped from 0 to 7 V. This voltage is reduced by a 100:1 voltage divider before it is applied to the excitation piezo. For each amplitude, 512 traces of *V*_sig_ are taken and averaged, where the start of sampling is triggered by the zero crossing of the low-noise sinusoidal cantilever excitation voltage signal recorded on the second oscilloscope channel.

For data evaluation, a simplified form of Equation 5 is fitted to the averaged trace for each amplitude. In the fit function of Equation 6, linearly depending parameters are gathered into one


[6] $$V_{\text{sig}}=V_{\text{DC}}+V_{0}\sin\left\{\frac{4\pi }{\lambda }\left[D-A\cdot \sin\left(2\pi f_{\text{s}}t-\varphi \right)\right]-\frac{\pi }{2}\right\},$$

where *V*_DC_ represents the constant part of the interferometer signal voltage, *V*_0_ the voltage amplitude of the interference signal oscillation, *D* = *d*_0_ + *d*_err_ − *q*_s_ the actual distance of the center of oscillation from the fiber end, *f*_s_ the frequency referenced to the time base of the oscilloscope, and φ a phase factor covering any phase shift introduced by the electronics in the signal path. The time dependence of *S*_loss_ is neglected as it is of minute influence on the amplitudes used here. However, for experiments with a very large amplitude, it is expected to influence the interference signal.

**Figure 3:**
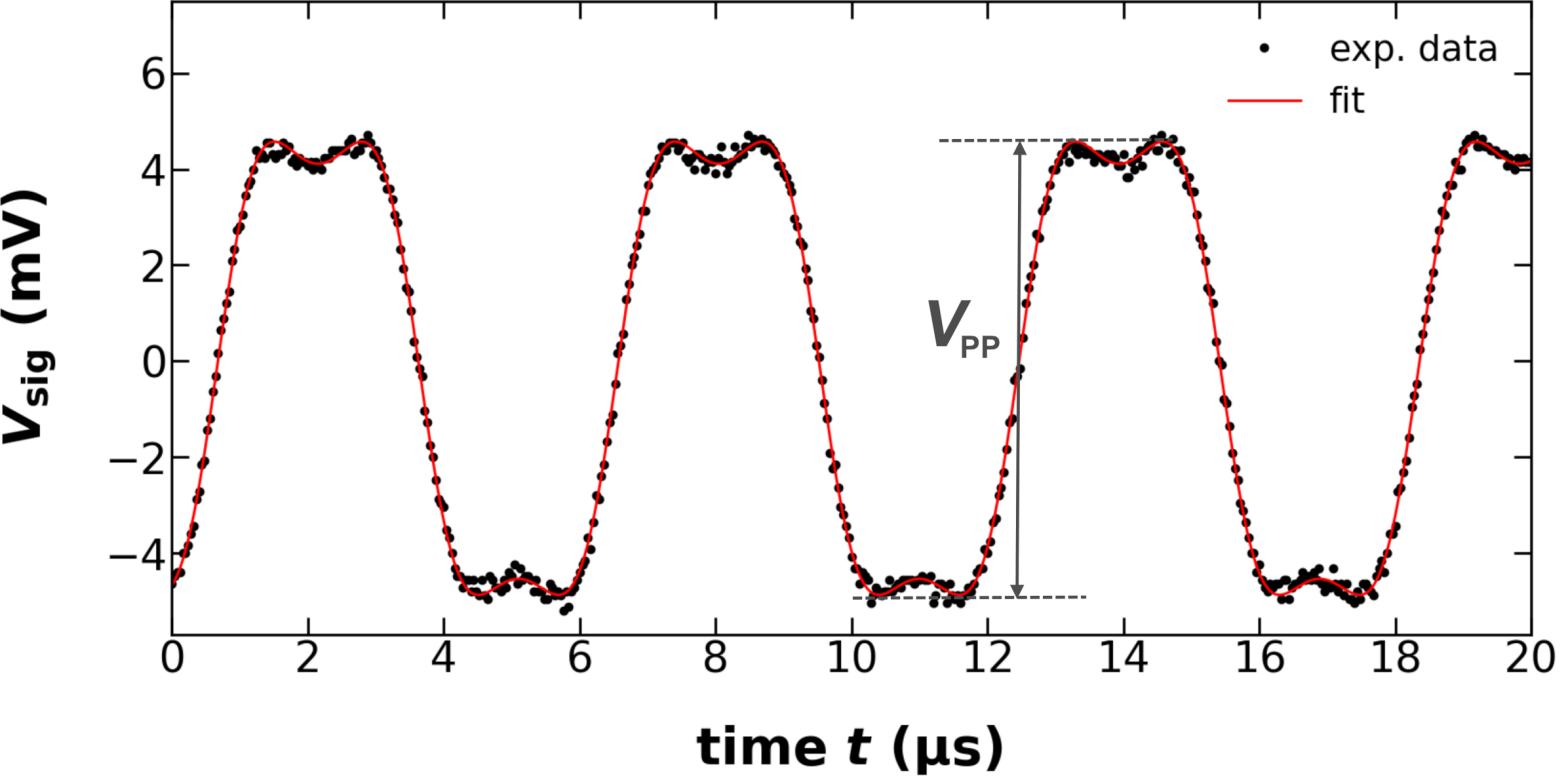
Fit of the model for the interferometer signal voltage *V*_sig_ according to Equation 6 to experimental data. The cantilever excitation piezo voltage amplitude is *V*_exc_ = 4.25 V corresponding to an amplitude *A* = 86.61 nm.

**Figure 4:**
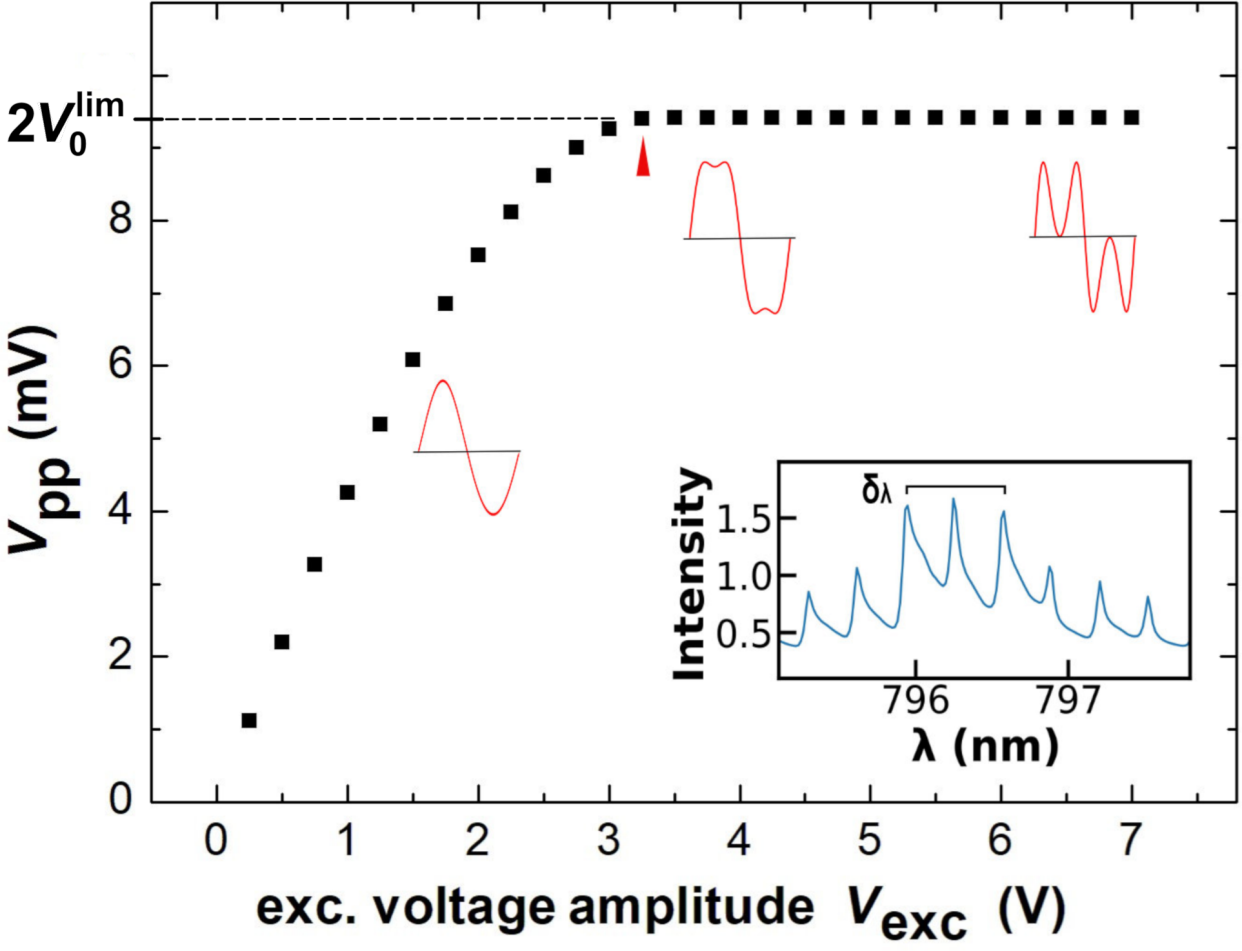
Peak-to-peak amplitude *V*_pp_ of *V*_sig_ (see Figure 3) as a function of the cantilever excitation voltage amplitude *V*_exc_. The red arrow points to the onset of saturation. The insets show three typical waveforms for *I*_M_(*t*) (*V*_exc_ = 1.75, 4, and 7 V) and the central part of the laser diode mode spectrum, with the wavelength representing the vacuum wavelength.

We find that Equation 6 fits the experimental data for all amplitudes perfectly, as demonstrated for one example in Figure 3. However, for lower amplitudes, the fit does not yield physically meaningful results because of the mutual dependence of the parameters *V*_0_, *A*, and φ. We find, for instance, that the fit value of *V*_0_ exhibits a dependence on *V*_exc_, while it is evident from Equation 5 that *V*_0_ should be a constant solely determined by system parameters. To yield the correct value 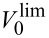, we plot the peak-to-peak amplitude *V*_pp_ of the *V*_sig_ fit curve (see Figure 3) as a function of *V*_exc_, as shown in Figure 4. We find that *V*_pp_ first rises with amplitude and then saturates at the amplitude limit 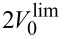 (red arrow in Figure 4). A parameter that can reliably be deduced from the fit is *f*_s_ as this is the characteristic fundamental frequency of the signal. In the second step of data evaluation, we perform a fit of the same fit function to the same experimental data, however, with a reduced number of fit parameters. In this fit, 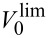 and *f*_s_ are taken over as fixed values from the first fit, while the other parameters are treated as free fit parameters. This two-step procedure allows us to determine all signal parameters with high accuracy.

As the interferometric method is perfectly suited for the calibration of the cantilever oscillation amplitude, we exemplify the fit procedure and accuracy limits for the fit parameter *A*. Amplitude calibration means to relate the cantilever oscillation amplitude *A* to the voltage *V*_exc_ to yield the calibration factor *S* = *A*/*V*_exc_ [18]. An accurate calibration is essential for quantitative NC-AFM; therefore, various methods have been suggested to determine the calibration factor *S* [10,19–22]. There is a simple and rough, but commonly used, method of calibration of the cantilever displacement by an interferometer, which is based on the measurement shown in Figure 4. This method uses just the data point for the excitation amplitude *V*_exc_(*A* = λ/8), where saturation in *V*_pp_ occurs (red arrow in Figure 4), indicating that the oscillation exactly covers one fringe with −λ/8 ≤ *q* ≤ + λ/8. For the experiment discussed here, such calibration yields *S* = 20.38 nm/V. However, from Figure 4 it is clear that the precision of this value is limited as the λ/8 point is not well defined.

**Figure 5:**
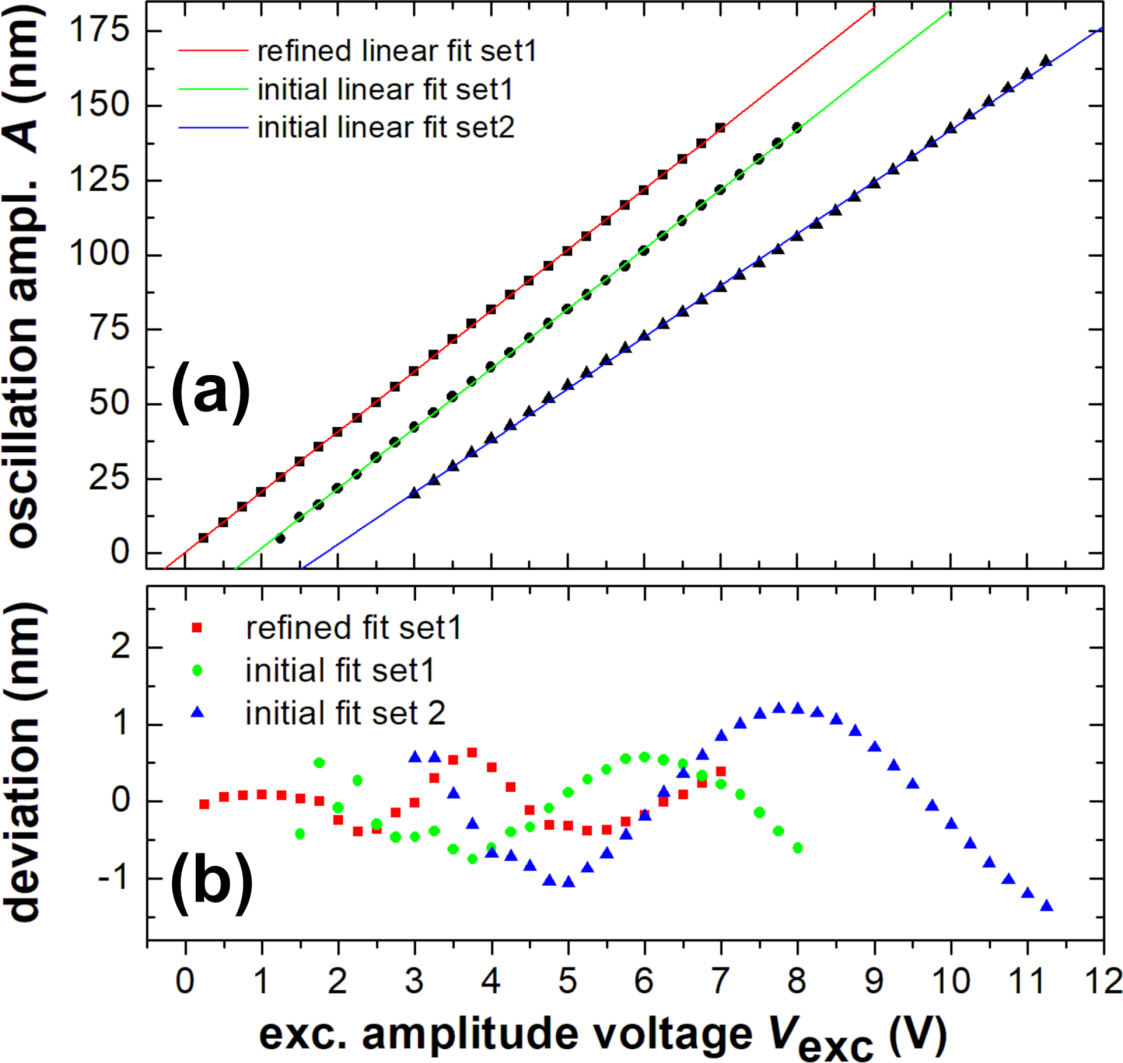
(a) The cantilever oscillation amplitude *A* is derived from the linear fit of Equation 6 to experimental time traces *V*_sig_(*t*) as a function of the excitation voltage amplitude *V*_exc_ (squares, circles, and triangles). Straight lines are linear fits of *A*(*V*_exc_) data taken in two different measurement runs and one that underwent two different analysis procedures. (b) Residuals of the oscillation amplitudes with respect to the linear fit. Note, that the green and blue data are shifted by 1 V along the *V*_exc_ axis for better visibility of the graphs.

Figure 5 illustrates the enhancement in accuracy that can be achieved by applying the two-step fit procedure for data analysis. In this plot of *A*(*V*_exc_), measurements taken at all amplitudes are included and fitted by a straight line. The green and blue curves represent measurements taken over two days, where the optical fiber has been re-adjusted slightly between the measurements. The curves (circle and triangle) represent data analyzed by a single fit, where the green curve represents the same data as those shown in Figure 4. Both measurements yield a linear behavior, however, with a somewhat different slope and, therefore, different calibration factors, which is due to the fiber re-adjustment. The residuals plotted in the lower part of the figure demonstrate that measurements are free of any significant noise [23]; however, we find a smooth undulation of the experimental values around zero, which stems from the residual mutual dependence of the fit parameters. The red curve (squares) represents the fit results for the data from the green curve treated with the two-step procedure. The analysis of the residuals reveals that the second step of data processing reduces, but cannot fully remove, the undulation.

At first sight, the undulation as a systematic error appears as the major limitation for the accuracy in determining the calibration factor *S*. An extended analysis of several sets of data covering a large range of amplitudes yields, however, that the effect of the undulation can be reduced to a negligible effect by a proper choice of the analyzed range of amplitudes. This is achieved by restricting the analysis to a range of amplitudes, where the undulating behavior yields a compensation of positive and negative deviations from the straight line. To obtain limits for the precision and accuracy of the result for the amplitude calibration factor, we consider four contributions to the error in *S*, which are expressed in the following formula of error propagation for the linear fit [24]:


[7] $$\delta_{\text{S}}=S\sqrt{{\left(\frac{\delta_{V_{\text{exc}}}}{V_{\text{exc}}}\right)}^{2}+{\left(\frac{\delta_{A}}{A}\right)}^{2}+{\left(\frac{\delta_{\lambda }}{\lambda }\right)}^{2}+{\left(\frac{\delta_{l_{\text{c}}}}{l_{\text{c}}}\right)}^{2}},$$

where 
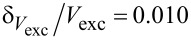
 is the excitation voltage output uncertainty according to the HF2 specification. To take care of the systematic error due to the dependency in fit parameters, we determine δ*_A_*/*A* = 0.0004 as the mean of the residuals in *A* divided by the mean value of *A*, determined as an oscillation amplitude error. The relative error in the wavelength measurement is δ_λ_/λ = 0.00075 as detailed below. The error in the adjustment of the light spot on the cantilever with length *l*_c_ is the positioning error δ*l*_c_ = 5 μm, which is the distance between the laser spot position and the tip position as illustrated in Figure 1. This error is estimated by visual inspection of a CCD camera image of the fiber–cantilever gap, and we find 
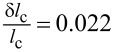
. To estimate the wavelength error δ_λ_, we performed a careful measurement of the laser diode light wavelength λ with a spectrograph (Acton series SP-2500i-2556, Princeton Instruments, USA), which has been calibrated by 40 atomic lines distributed over the entire visible spectrum to yield an accurate value for the wavelength at a spectral resolution of 0.050 nm. As evident from the multimode spectrum of the laser diode light source shown in the inset of Figure 4, the spectrum is dominated by three modes with a center at the vacuum wavelength λ = 796.49 nm. Assuming that interference occurs in the optical fiber, we calculate the laser wavelength in the fiber with *n* = 1.45 as λ_f_ = 549.3 nm for oscillation amplitude calibration. We take the spectral distance of the two neighboring lines as a conservative estimate for the wavelength error δ_λ_ = 0.41 nm. Note, that the errors 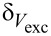 and δ_A_ are not independent variables. We treat them separately as 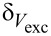 is a statistical error, while δ_A_ represents an additional systematic error due to the residuals in the linear fit of *A*(*V*_exc_). Taking these error margins into account, we obtain the final result for the amplitude calibration factor *S* = (20.30 ± 0.49) nm/V.

## Conclusion

In summary, we derived a model for the description of the time domain signal of a Michelson-type interferometer used to measure the displacement of a (quasi) harmonically oscillating microcantilever in an NC-AFM. The analysis demonstrates that the interferometer signal is a non-trivial function of the cantilever excitation, where increasing excitation amplitude is translated into increasing non-linearity and complexity of the response signal. A fit of the derived response function to experimental data yields excellent results for all system parameters. However, care has to be taken to minimize systematic errors resulting from the mutual dependence of fit parameters. The method specifically allows one to determine the cantilever oscillation amplitude calibration factor with a 2.4% relative error. This is way better than what can be achieved with a calibration based on the widely used γ-method [19]. We recently investigated the precision of the γ-method in detail and found that it is intrinsically prone to systematic error, where an error of 15% may result under realistic experimental conditions [25].

The strength of the interferometric calibration is the high precision that can be achieved as the calibration of the amplitude can be traced to the light wavelength, which can be measured most precisely and accurately. The error analysis shows that the weakest point relevant for NC-AFM measurements is the accurate positioning of the light beam at the position of the tip. In experiments, as introduced here, noise is not a limiting factor for the quantitative evaluation of the interferometric signal, and there is still room for improvement by optimising the experimental setup.

## Data Availability

Data available upon request from the authors.

## References

[1] Yang S, Zhang G (2018). Meas Sci Technol.

[2] Bond C, Brown D, Freise A, Strain K A (2016). Living Rev Relativ.

[3] Buikema A (2020). Phys Rev D.

[4] Heinze J, Danzmann K, Willke B, Vahlbruch H (2022). Phys Rev Lett.

[5] Rugar D, Mamin H J, Erlandsson R, Stern J E, Terris B D (1988). Rev Sci Instrum.

[6] Hoogenboom B W, Frederix P L T M, Yang J L, Martin S, Pellmont Y, Steinacher M, Zäch S, Langenbach E, Heimbeck H-J, Engel A (2005). Appl Phys Lett.

[7] Hoogenboom B W, Frederix P L T M, Fotiadis D, Hug H J, Engel A (2008). Nanotechnology.

[8] Morita K, Sugimoto Y, Sasagawa Y, Abe M, Morita S (2010). Nanotechnology.

[9] Karci O, Dede M, Oral A (2014). Rev Sci Instrum.

[10] Çelik Ü, Karcı Ö, Uysallı Y, Özer H Ö, Oral A (2017). Rev Sci Instrum.

[11] von Schmidsfeld A, Nörenberg T, Temmen M, Reichling M (2016). Beilstein J Nanotechnol.

[12] von Schmidsfeld A, Reichling M (2015). Appl Phys Lett.

[13] Söngen H, Bechstein R, Kühnle A (2017). J Phys: Condens Matter.

[14] Tröger L (2009). Aufbau eines Tieftemperatur-Rasterkraftmikroskopes.

[15] Lübbe J, Tröger L, Torbrügge S, Bechstein R, Richter C, Kühnle A, Reichling M (2010). Meas Sci Technol.

[16] Drevniok B, Paul W M P, Hairsine K R, McLean A B (2012). Rev Sci Instrum.

[17] Moheiman S O R (2008). Rev Sci Instrum.

[18] Rahe P, Heile D, Olbrich R, Reichling M (2022). Beilstein J Nanotechnol.

[19] Simon G H, Heyde M, Rust H-P (2007). Nanotechnology.

[20] Sugimoto Y, Nakajima Y, Sawada D, Morita K-i, Abe M, Morita S (2010). Phys Rev B.

[21] Martínez J F G, Nieto-Carvajal I, Colchero J (2013). Nanotechnology.

[22] Dagdeviren O E, Miyahara Y, Mascaro A, Grütter P (2019). Rev Sci Instrum.

[23] Lübbe J, Temmen M, Rode S, Rahe P, Kühnle A, Reichling M (2013). Beilstein J Nanotechnol.

[24] Hughes I G, Hase T P A (2009). Measurements And Their Uncertainties.

[25] Heile D, Olbrich R, Reichling M, Rahe P (2021). Phys Rev B.

